# Anisotropic Magnetoelectric Effect in a Planar Heterostructure Comprising Piezoelectric Ceramics and Magnetostrictive Fibrous Composite

**DOI:** 10.3390/ma12193228

**Published:** 2019-10-02

**Authors:** Yuri Fetisov, Dmitri Chashin, Dmitri Saveliev, Leonid Fetisov, Mikhail Shamonin

**Affiliations:** 1 Research and Education Center “Magnetoelectric Materials and Devices”, MIREA–Russian Technological University, 119454 Moscow, Russia; chashindv@yandex.ru (D.C.); dimsav94@gmail.com (D.S.); fetisovl@yandex.ru (L.F.); 2 East Bavarian Centre for Intelligent Materials (EBACIM), Ostbayerische Technische Hochschule (OTH) Regensburg, D-93053 Regensburg, Germany

**Keywords:** Magnetoelectric effect, magnetostrictive fiber, piezoelectric ceramic material, heterostructure, composite, frequency doubling

## Abstract

The direct magnetoelectric (ME) effect is investigated in a planar structure comprising mechanically coupled layers of a magnetostrictive fibrous composite (MFC) and a piezoelectric ceramics (lead zirconate titanate, PZT). The MFC is an array of Ni-wires with a diameter of 200 μm that are aligned parallel to each other in a single layer. The wires are separated by a distance of 250 or 500 μm and fixed in a polyamide matrix. The structure was placed in a tangential constant field ***H*** and was excited by an alternating magnetic field ***h*** parallel to ***H***, while the voltage generated by the PZT layer was measured. The resulting field dependences of the magnetization *M*(*H*) and the magnetostriction *λ*(*H*) were determined by the orientation of the field ***H*** in the plane of the structure and the distance between the Ni-wires. The ME coupling coefficient of the structure decreased from 4.8 to 0.25 V/A when the orientation of ***H*** was changed from parallel to perpendicular to Ni-wires. With an increase in the excitation field amplitude *h*, a nonlinear ME effect in the output voltage, namely frequency doubling, was observed. The frequency and field dependences of the efficiency of the ME transduction in the MFC-piezoelectric heterostructure are well described by the existing theory.

## 1. Introduction

Magnetoelectric (ME) effects in composite heterostructures consisting of mechanically coupled ferromagnetic (FM) and piezoelectric (PE) layers have been intensively investigated because of their promising applications in highly sensitive magnetic field sensors [1], electrically controlled devices for radio signals processing [2], and self-sufficient sources of electrical energy [3]. ME effects accomplish the mutual transformation of magnetic and electric fields in solid-state structures. The direct ME effect leads to the generation of an alternating electric field ***e*** in the PE layer of the structure under the influence of an alternating magnetic field ***h***, while the converse ME effect causes the modulation of magnetization in the FM layer of the structure as the result of an alternating electric field. Both effects arise due to the interplay of magnetostriction of the FM layer and piezoelectricity in the PE layer caused by the mechanical coupling between the layers [4].

The direct ME effect in composite heterostructures is characterized by the coefficient $\alpha_{E}=e/h=u/(h\cdot a_{p}^{})$, where *u* is the amplitude of the voltage generated between the electrodes of the PE layer, *h* is the amplitude of the magnetic excitation field, *a*_p_ is the thickness of the PE layer. It has been shown that the magnitude of the coefficient is proportional to the piezomagnetic modulus *q* of the FM layer and the PE modulus *d* of the PE layer in the heterostructure [5]. In turn, the piezo-moduli depend on external fields, which makes it possible to control the efficiency of the ME transformation using an external constant magnetic field ***H*** [6] or a constant electric field ***E*** applied to the PE layer [7]. Usually, in order to use weak control fields and avoid demagnetization effects, the control magnetic field is applied parallel to the structure plane and the excitation field (***H*** // ***h***).

The ME effect is isotropic in heterostructures containing isotropic FM and PE layers, i.e., its characteristics do not depend on the orientation of the field ***H*** in the structure plane. However, for a number of applications, e.g., sensors sensitive to the magnetic field orientation, structures with an anisotropic ME effect are required. To fabricate such structures, one can use layers with anisotropic magnetostriction or an anisotropic PE effect. The natural way is to employ the crystallographic anisotropy of materials. An anisotropic ME effect has been observed in structures with FM layers of single-crystalline CoFe_2_O_4_ [8] and PE layers of single crystals of lead magnesium niobate-lead titanate (PMN-PT) [9], LiNbO_3_ and GaPO_4_ [10]. It has been shown that the adjustment of the layer orientation in structures with single crystals—CoFe_2_O_4_–BaTiO_3_, for example—can increase the ME coefficient [11]. Magnetostriction anisotropy can be artificially created during material fabrication. Thus, the anisotropic ME effect was observed in a heterostructure with a layer of isotropic soft relaxor type lead zirconate titanate (PZT) ceramics (navy type II) and a layer of the FM CoFe_2_O_4_ ceramic material, in which the uniaxial magnetic anisotropy was induced by pressure during fabrication [12].

The anisotropy of the ME effect has been observed in heterostructures with layers of a PE fibrous composite (PFC), developed by the Smart Material Corporation (Sarasota, FL, USA) [13,14], and FM layers of an isotropic magnetostrictive material. PFC is an array of fibers made of a PE lead zirconate titanate (PZT) ceramic with a transverse diameter of 250 μm arranged in a single layer parallel to each other and fixed in a polyamide matrix. PFC generates electrical voltage only when it is deformed along the axis of the PZT fibers. The large values of the PE modulus, flexibility and low material costs have enabled the wide use of PFC in structures used for the fabrication of ME magnetic field sensors.

In the present work, a highly anisotropic ME effect was observed and investigated in a composite heterostructure, in which a new material, magnetostrictive fibrous composite (MFC), was employed as the anisotropic FM layer, and an isotropic PZT ceramic material was used as the PE layer. The MFC is an array of FM wires arranged in a single row at some distance from each other and fixed in a polymer matrix. Due to its geometrical effect, MFC was deformed in different ways when a constant magnetic field ***H*** was applied along or across the axis of the FM wires. This led to the anisotropy of the MFC characteristics and, as a result, to a strong anisotropy of the ME characteristics when the constant magnetic field was rotated in the heterostructure’s plane.

In the following section of the paper, the fabrication technology of the MFC and the technique for measuring the characteristics of the MFC and the composite structure as a whole are described. The third section presents the measured magnetic and magnetostrictive characteristics of the MFC. In what follows, the frequency, field and amplitude characteristics of anisotropic linear and nonlinear ME effects in the fabricated heterostructure are described. The obtained results are discussed and analyzed in the fourth section of the paper. In the concluding section, the results of research and prospects for the usage of the anisotropic ME effect in the described heterostructure are summarized.

## 2. Materials and Methods 

**Figure 1:**
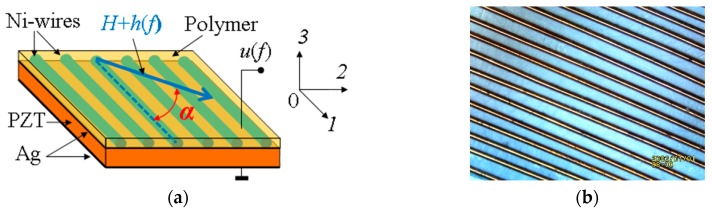
(**a**) Schematic representation of the magnetostrictive fibrous composite-piezoelectric (MFC-PE) planar structure: Ni stands for the nickel wires, PZT (lead zirconate titanate) is the piezoelectric plate, Ag is the electrodes, and Polymer is the polymer layer. α denotes the angle between the wires and the applied magnetic field; (**b**) MFC fragment with Ni-wires with a diameter of 200 μm located at a distance of 500 μm.

The investigated heterostructure is shown schematically in Figure 1a. The structure consists of an MFC and a plate made of PZT with the chemical formula PbZr_0.52_Ti_0.48_O_3_. The MFC was an arrangement of wires with a diameter of 200 μm made of the electrolytically purified Ni, placed in a single layer parallel to each other, and embedded into a polymer matrix. The MFC dimensions were 10 × 10 mm, with a thickness *a*_m_ of about 205 μm. Two MFC specimens were fabricated, and each had a period of Ni-wire arrangement *T*_1_ of 250 μm and a period *T*_2_ of 500 μm. As an example, Figure 1b presents, on the enlarged scale, a fragment of the MFC with the period *T*_2_ before the pouring of the polymer. The PZT plate had the planar dimensions of 10 × 10 mm and a thickness *a*_p_ of 200 μm. The Ag electrodes, with a thickness of about 2 μm, were deposited on the plate surface, and the PE plate was poled perpendicular to its plane. The PE modulus *d*_31_ of the PZT plate was 175 pm/V. The MFC and the PZT plate were mechanically bonded to each other with epoxy glue under a press. For investigations, two structures containing an MFC with different periods were fabricated. According to the accepted classification [15], the described ME heterostructure belongs to composite materials with the connectivity of the “1–2”-type. 

**Figure 2:**
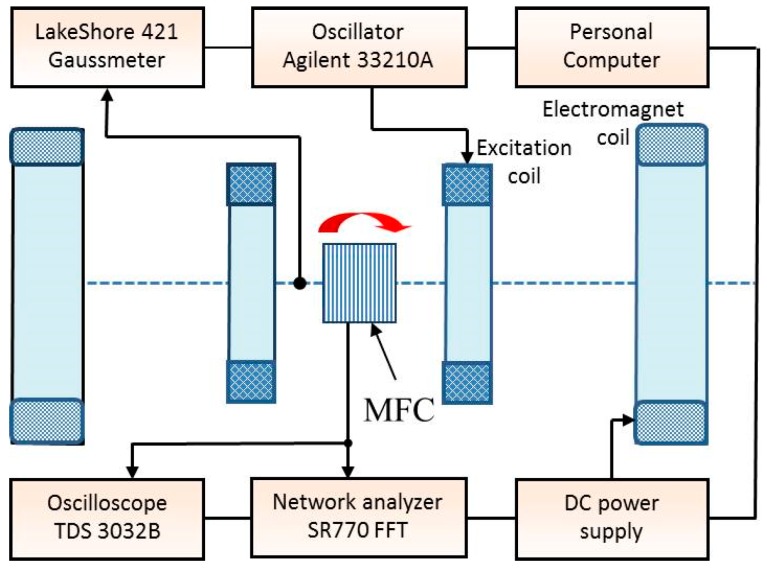
Schematic diagram of the experimental setup.

The schematic diagram of the experimental setup is shown in Figure 2. For the measurements of characteristics, the structures were horizontally suspended between the poles of an electromagnet on thin conductors soldered to the corners of the PZT plate. The electromagnet generated a constant uniform field *H* = |***H***|= 0–200 kA/m, directed tangentially to the plane of the structure, with the variable inclination with respect to the MFC wires.

Simultaneously, an excitation magnetic field with an amplitude *h* = 0–278.5 A/m and a frequency *f* = 0–30 kHz was applied in the same direction using a Helmholtz coil fed by a signal generator (Agilent 33210A, Agilent Technologies, Santa Clara, USA). The amplitude of the alternating voltage *u*(*f*) generated between the PZT layer electrodes was recorded using a TDS 3032B oscilloscope (Tektronix, Beaverton, USA). The frequency spectrum of the voltage was measured using a low-frequency SR770 FFT Network Analyzer (Stanford Research Systems, Sunnyvale, USA). The magnetic field was measured with a resolution of 7.96 A/m (0.1 Oe) using a gaussmeter (Lake Shore 421) (Lake Shore Cryotronics, Westerville, USA). The uncertainty of field measurements with the magnetometer was ±1%. The setup allowed one to record the dependence of the voltage *u* on the frequency *f*, ***h*** and ***H***-fields in the automated mode using a personal computer. The magnetization curves of the structure *M*(*H*) were measured in the field range of between 0 and 398 kA/m using a vibrating sample magnetometer (Lake Shore 7407). The field dependences of the magnetostriction *λ*(*H*) were measured on an automated setup in fields up to 79.6 kA/m with an accuracy *δλ* ≈ ±1 × 10^−6^ using a strain gauge glued onto the MFC surface [16]. 

## 3. Results

### 3.1. MFC Magnetization Curves

**Figure 3:**
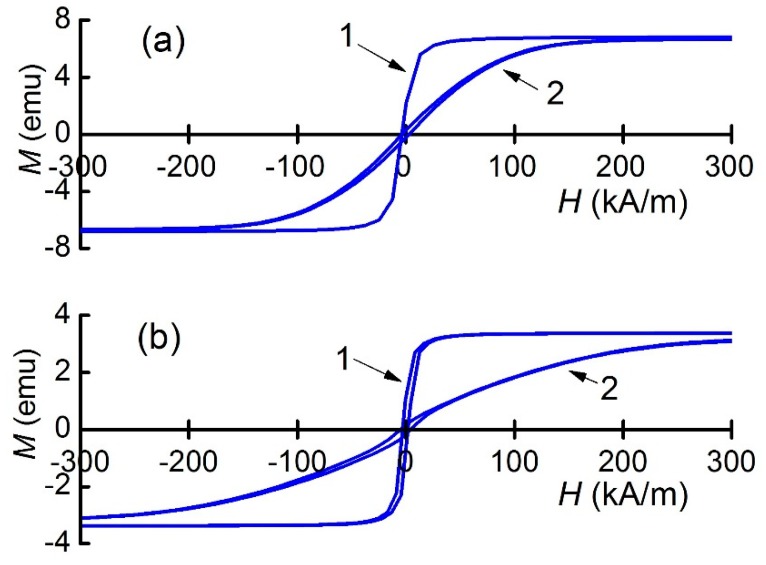
Magnetization curves of MFCs with the spatial period of (**a**) *T*_1_ = 250 μm and (**b**) *T*_2_ = 500 μm for the constant magnetic field ***H*** oriented along (1) and perpendicular to (2) to Ni-wires.

At the first stage, the magnetization curves *M*(*H*) of both fabricated MFC samples were measured for the magnetizing field ***H*** in the sample’s plane parallel and perpendicular to the Ni-wires. Figure 3a shows the obtained curves for the structure with a period *T*_1_ = 250 μm. When the field ***H*** was oriented parallel to the wires, magnetization *M* was saturated in small fields *H*_S_ ~ 50 kA/m; when when the field was oriented perpendicular to the wires, magnetic saturation happened at *H*_S_ ~ 240 kA/m. The coercive force was *H*_C_ ≈ 1.59 kA/m, when the field ***H*** was applied parallel to the wires, and *H*_C_ ≈ 3.58 kA/m when the field was directed perpendicular to the wires. Figure 3b presents similar dependences for the MFC with a period *T*_2_ = 500 μm. When the field ***H*** was oriented parallel to the wires, *M* was saturated in the same field *H*_S_ ~ 50 kA/m, and when the field was oriented perpendicular to the wires, the magnetic saturation was observed in a much larger field *H*_S_ ~ 400 kA/m. The coercive force *H*_C_ was increased to ≈2.38 kA/m for the field orientation parallel to the wires and to *H*_C_ ≈ 4.77 kA/m for the field directed perpendicular to the wires. As expected, the saturation magnetization of the MFC was decreased approximately twofold with a twofold increase in the period and a corresponding decrease in the total mass of Ni-wires.

The measurement data demonstrate a strong anisotropy of the dependence of magnetization *M* on the orientation of magnetic field ***H*** in the MFC plane and the ability to control the slope of *M*(*H*) for a given orientation of the field by changing the distance between the Ni wires in the MFC. 

### 3.2. MFC Magnetostriction

**Figure 4:**
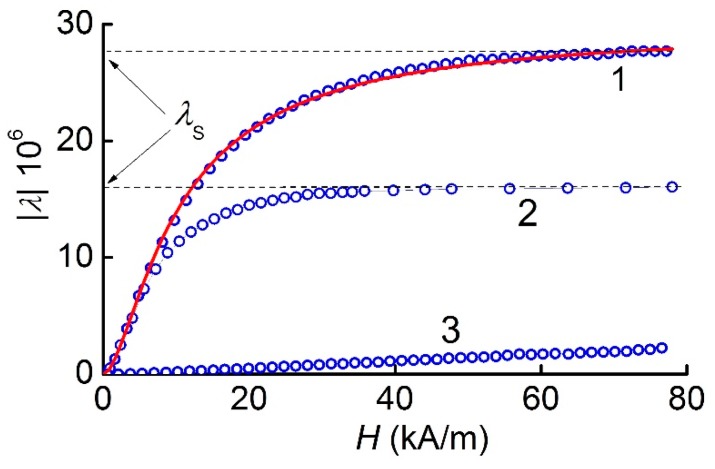
Dependence of magnetostriction *λ* on the field magnitude *H* for: 1-MFC with a period *T*_1_ of 250 μm, magnetized along the wires; 2-MFC with a period *T*_2_ of 500 μm, magnetized along the wires; and 3-MFC with a period *T*_1_ of 250 μm, magnetized across the wires. The solid curve is the approximation of magnetostriction (*λ*(*H*)) by Equation (5).

Figure 4 depicts the measured dependences of the magnetostrictive deformation for both MFC samples. The magnetostriction of nickel was negative. Therefore, for convenience, the vertical axis of Figure 4 shows the absolute value. It can be seen that when the MFC sample with *T*_1_ = 250 μm was magnetized along the wires, the magnetostriction reached a maximum value of *λ*_S_ ≈ −28 × 10^−6^ in the saturation field *H*_S_ ≈ 63.7 kA/m; meanwhile, the magnetostriction was saturated at a level of λ_S_ ≈ −16 × 10^−6^ in the saturation field *H*_S_ ≈ 47.7 kA/m for the MFC with a period *T*_2_ of 500 μm. Moreover, in the region of small fields (*H* < 8 kA/m), the curves almost overlapped each other.

When the MFC with a period *T*_1_ of 250 μm was magnetized perpendicular to the Ni wires, the maximum magnetostriction reached a value of approximately 2 × 10^−6^ in fields of about 50 kA/m. For the sample with a period *T*_2_ of 500 μm, when the magnetic field was directed perpendicular to the Ni-wires, the magnetostriction did not exceed the measurement uncertainty of ±1 × 10^−6^.

Thus, there was a strong anisotropy of the *λ*(*H*)-dependence in the MFC plane. At the same time, the saturation magnetostriction *λ*_S_ for the MFC with a smaller period *T*_1_ and orientation of the ***H***-field along the Ni-wires was slightly less than the saturation magnetostriction of a solid Ni plate [17], and it was further reduced twofold when the period was doubled.

Notice that the saturation fields *H*_S_ of the magnetostriction coincided with the saturation field of the magnetization.

### 3.3. Frequency Dependence of the ME Voltage

**Figure 5:**
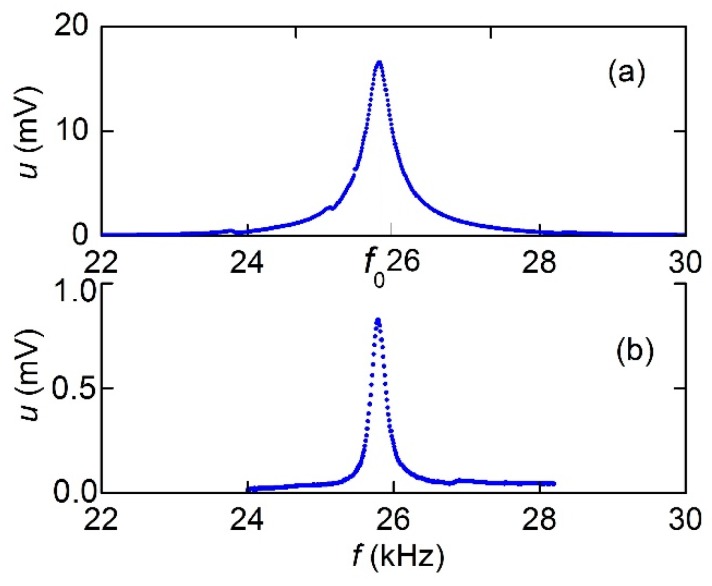
Dependence of the magnetoelectric (ME) voltage *u* on the excitation frequency *f* in the MFC-PZT structure with a period *T*_1_ = 250 μm and the magnetic field directed (**a**) parallel and (**b**) perpendicular to Ni-wires.

At the next stage, the characteristics of the direct ME effect were investigated. Figure 5 presents the dependences of the amplitude of the ME voltage *u* on the frequency of the excitation field for the MFC-PZT structure with a period of *T*_1_ = 250 μm in the magnetic field ***H*** directed along and perpendicular to the Ni-wires and an excitation field amplitude *h* of 17.5 A/m. For both field orientations, the dependences *u*(*f*) showed resonance peaks with the same resonance frequency *f*_0_ ≈ 25.8 kHz and the quality factor *Q* of ≈ 90 when the field was directed along the wires and ≈ 150 in the perpendicular direction.

When the structure was magnetized along the MFC wires (Figure 5a), the maximum value of the voltage was observed at *H*_m_ ≈ 3.18 kA/m. When the field was oriented perpendicular to the wires (Figure 5b), the maximum was observed at *H*_m_ ≈ 47.7 kA/m. Moreover, the maximum voltage amplitude decreased from *u*_m_ ≈ 16.5 to ≈ 0.8 mV when the orientation of ***H*** was changed from parallel to perpendicular with respect to the wires. As is shown below, the peak corresponded to the excitation of the lowest mode of flexural vibrations in the structure. The resonance of planar oscillations of the structure, the frequency of which was estimated to be about 192 kHz, was not observed. Similar to Figure 5, the resonance dependence *u*(*f*) was also obtained for MFC-PZT structure with a period *T*_2_ of 500 μm. In this case, the resonance frequency was approximately 24 kHz, and the maximum amplitude of the voltage generated by this structure was about 1.4 times smaller than for *T*_1_ = 250 μm. 

### 3.4. Dependence of the ME Voltage on the DC Field

**Figure 6:**
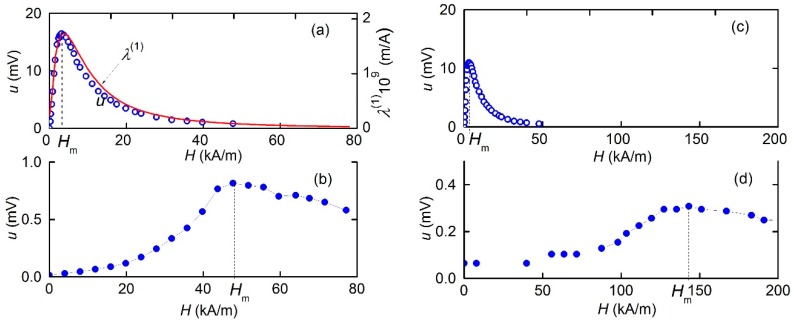
Dependence of the ME voltage *u* on the field magnitude *H* for the MFC-PZT structures with a period *T*_1_ = 250 μm (**a**,**b**) or *T*_2_ = 500 μm (**c**,**d**), magnetized either along (**a**,**c**) or across (**b**,**d**) the Ni-wires. The solid curve is the calculated field dependence of the piezomagnetic coefficient *λ*^(1)^(*H*).

Figure 6 presents the dependences of the ME voltage *u* generated by the MFC-PZT structure with a period *T*_1_ = 250 μm at the resonant frequency *f*_0_ on the constant field magnitude *H* at *h* = 17.5 A/m. When MFC was magnetized along Ni-wires (Figure 6a), the voltage initially grew linearly with *H*, reached a maximum *u*_m_ ≈ 16.5 mV at *H*_m_ ≈ 3.18 kA/m, and then asymptotically declined to zero because the Ni-wires became magnetically saturated. In the region of low fields (*H* < 1.59 kA/m), the responsivity of the structure to a constant field *u*/*H* was ≈ 7.54 × 10^−6^ V∙m/A. The shape of the *u*(*H*)-dependence slightly changed when the direction of the constant magnetic field was inverted. The magnitude of the coercive field was *H*_C_ ≈ 1.59 kA/m. For comparison, Figure 6a also shows the field dependence of the piezomagnetic coefficient *λ*^(1)^(*H*), calculated using Equation (5), which approximates the measured dependence of the magnetostriction *λ* on the field *H*. 

For the case when the structure was magnetized perpendicular to Ni-wires, the corresponding *u*(*H*)-dependence is shown in Figure 6b. The voltage was significantly lower, and *u* monotonously increased with increasing *H*, reached a maximum of approximately 0.8 mV at *H_m_* ≈ 48 kA/m, and then monotonously declined. Simultaneously, the coercive field increased to *H*_С_ ≈ 4.8 kA/m. The dependence shown in Figure 6 indicates a strong anisotropy of the ME effect in the plane of the structure.

Similar field dependences were also obtained for the MFC-PZT structure with a period *T*_2_ = 500 μm (Figure 6c,d). The maximum voltage for the magnetization of the structure along Ni-wires was observed approximately in the same field *H*_m_ ≈ 3.98 kA/m, and when the structure was magnetized perpendicular to the wires, the maximum voltage was observed in a larger field *H*_m_ ≈ 143 kA/m. When this structure was magnetized along the wires, the dependence of *u* on *H* was almost the same as for the structure with a period of 250 μm, since Curve 2 in Figure 4 is superimposed on Curve 1 in the field region *H* < 8 kA/m. When the structure was magnetized across the wires, a slightly smaller voltage amplitude was observed at *H*_m_ = 3.98 kA/m, while the maximum was shifted to a slightly larger field. Note that the curve in Figure 6d is almost at the detection limit of measurements, since the noise voltage was of the order of 0.1 mV.

**Figure 7:**
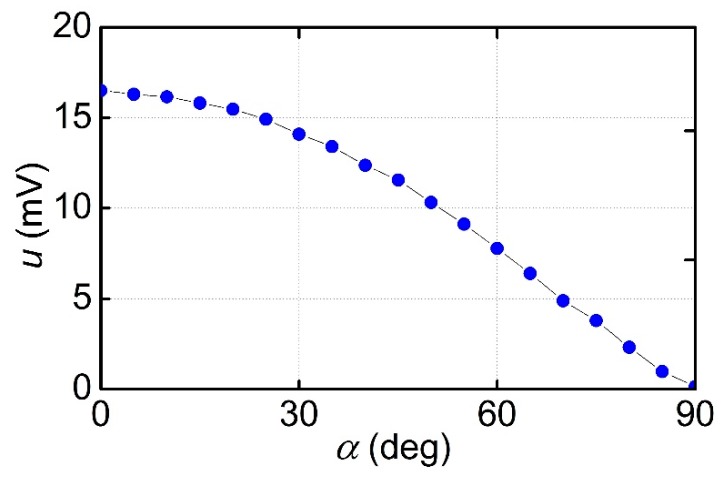
Dependence of the ME voltage at the resonance frequency with *H* = 3.18 kA/m and *h* = 17.5 A/m on the angle α between the ***H***-field and the Ni-wires.

Figure 7 demonstrates the angular dependence of the ME voltage at the resonance frequency for the MFC-PZT structure with a period *T*_1_ = 250 μm at a constant field *H*_m_ = 3.18 kA/m and *h* = 17.5 A/m. The orientation of the ***H***-field in the structure’s plane was moved from parallel to perpendicular with respect to the Ni-wires. The excitation field ***h*** remained parallel to ***H***. It can be seen that as the angle *α* increased, the voltage amplitude monotonously decreased from 16.5 to about 0.3 mV. Similar dependences were obtained for other values of *H* and rotation of the DC (direct current) field. The voltage values *u*(*α* = 0°) and *u*(*α* = 90°) can be found from Figure 5 at the corresponding field *H*. Note that in a structure with a continuous Ni layer, the ME voltage practically did not change with the variation of the ***H***-direction.

### 3.5. Dependence of the ME Voltage on the Excitation Field

**Figure 8:**
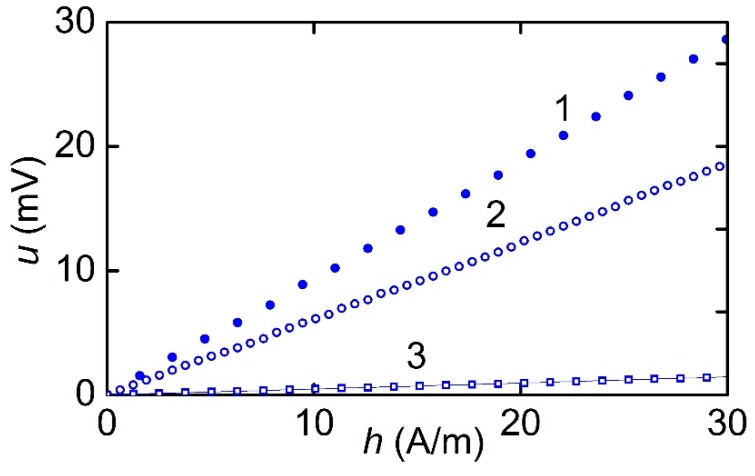
Dependence of the МE voltage *u* on the excitation field amplitude *h* for various MFC-PE structures: 1-*T*_1_ = 250 μm, magnetized along the wires; 2-*T*_2_ = 500 μm, magnetized along the wires; and 3-*T*_1_ = 250 μm, magnetized across the wires.

Figure 8 shows the dependences of the voltage amplitude *u* generated by MFC-PZT structures with a period *T*_1_ = 250 μm or *T*_2_ = 500 μm at the resonant frequency on the amplitude of the excitation field *h* when the structures were magnetized along (*H* = 3.18 kA/m) and perpendicular (*H* = 47.7 kA/m) to the Ni wires. All field dependencies of the voltage in this field region were linear. When the structures were magnetized along Ni wires, the maximum responsivity *u/h* of the structure with a period *T*_1_ of 250 μm to an AC (alternating current) field was approximately 0.94 mV∙m/A, and the maximum responsivity *u*/*h* of the structure with *T*_2_ = 500 μm was about 0.62 mV∙m/A. When magnetized across the Ni wires, the maximum responsivity of the structure with *T*_1_ = 250 μm was 45 μV∙m/A.

### 3.6. Nonlinear Frequency Doubling

With an increase in the amplitude of the excitation field *h* in the MFC-PZT structure with a period *T*_1_ of 250 μm and magnetization along the Ni-wires, nonlinear phenomena that are typical of composite structures were observed—resonant frequency doubling, in particular.

**Figure 9:**
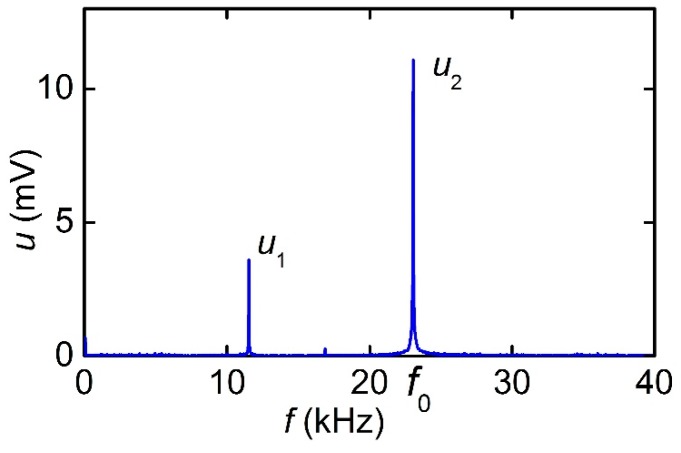
The frequency spectrum of the ME voltage generated by the MFC-PZT structure with a period *T*_1_ of 250 μm when magnetized along the wires by the *H*-field of approximately 40 A/m.

Figure 9 shows the frequency spectrum of the ME voltage when the structure was excited by an alternating field with an amplitude *h* of 279 A/m at a frequency *f*_1_ = *f*_0_/2 = 12.9 kHz, equal to the half of the resonant frequency. In the spectrum, besides the component at the excitation frequency, the second harmonic with a frequency of 25.8 kHz was visible, which was amplified by the acoustic resonance of the structure.

**Figure 10:**
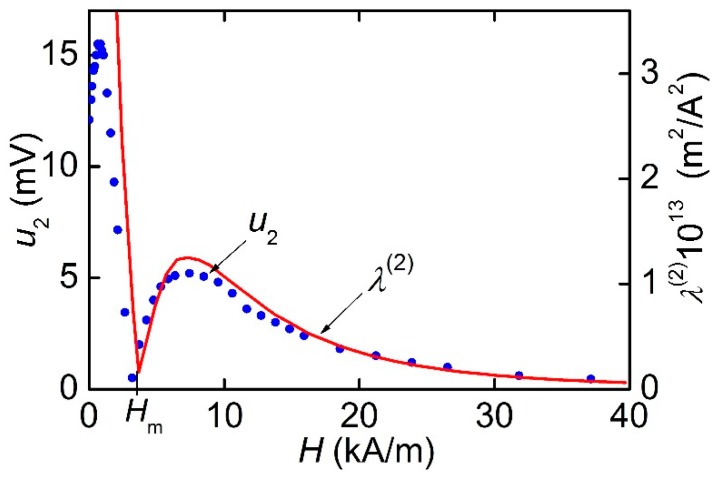
Dependence of the second harmonic amplitude *u*_2_ on the constant magnetic field *H* in the МFC-PZT structure with *T*_1_ = 250 μm. The solid curve is the calculated field dependence of the nonlinear piezomagnetic coefficient *λ*^(2)^(*H*).

Figure 10 presents the dependence of the second harmonic amplitude on the constant field magnitude *H* when the amplitude of the excitation field *h* was equal to 271 A/m. The amplitude of the harmonic *u*_2_ was about 12 mV in the absence of a constant field. It had the maximum value at *H* ≈ 640 A/m and dropped to almost zero in the field *H*_m_ ≈ 3.18 kA/m. Then, the second harmonic amplitude had a local maximum, and, finally, it monotonously declined to zero when the magnetostriction of the Ni-wires was saturated. The field for the minimum value of the second harmonic amplitude in Figure 10 coincided with the field for the maximum of the first harmonic in Figure 6. For comparison, Figure 10 also demonstrates the field dependence of the nonlinear piezomagnetic coefficient *λ*^(2)^(*H*), calculated using Equation (5), which approximates measured dependence *λ*(*H*).

**Figure 11:**
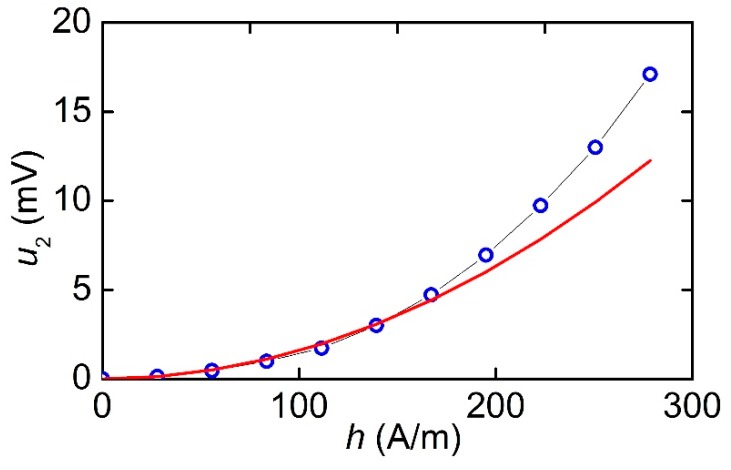
Dependence of the second harmonic amplitude *u*_2_ on the excitation field amplitude *h* at *H* = 0 for the МFC-PZT structure with *T*_1_ = 250 μm. The solid curve is the quadratic approximation of experimental data.

Figure 11 shows the dependence of the amplitude of the second harmonic on the amplitude of the excitation field *h* at *H* = 0. In the region of small amplitudes *h* < 159 A/m, the dependence could be well described by a quadratic function *u*_2_~*h*^2^. With the field *h* was oriented across the Ni-wires in the structure with a period *T*_1_ of 250 μm, no doubling of the output voltage frequency was observed. Frequency doubling with lower efficiency was also found in the MFC-PZT structure with a period *T*_2_ of 500 μm when it was magnetized along the Ni-wires.

## 4. Discussion

The measurement results demonstrate the strong anisotropy of the magnetization *M*, the magnetostriction *λ* and the ME coefficient α_E_ in MFC-PZT structures with a change in the orientation of the constant field ***H*** in the plane of the structures. In this case, the origin of magnetic anisotropy was the geometrical alignment of Ni-wires in one direction. Bakaev et al. [18] calculated the effective magnetic permeability of randomly inhomogeneous fibrous ferromagnetic composites both along and across fibers. They demonstrated that such a material can be more easily magnetized along fibers than across them. Such an effect can be observed in Figure 3, where the saturation field *H*_S_ is shown to be significantly larger, and the slope of the *M*(*H*) curves (i.e., the apparent magnetic susceptibility) is shown to be significantly smaller when the magnetization of the MFC occurred perpendicular to the axes of Ni-wires. 

It is known [19,20] that magnetic field inside a ferromagnet *H*_in_ is connected to the external magnetizing field *H* by Equation (1):

(1) $$H_{\mathrm{in}}=H-N\cdot M(H_{\mathrm{in}}),$$

where *N* is the demagnetizing factor depending on the sample’s shape and *M*(*H*_in_) is the material magnetization. For an infinitely long FM rod magnetized along the axis “1” (see Figure 1a), the demagnetizing factor is zero, and for a rod magnetized in the transverse direction, i.e., along the axis “2”, the demagnetizing factor is 0.5. Thus, in a long longitudinally magnetized FM rod, the internal magnetic field is approximately equal to the external field, while in a transversely magnetized rod, the internal field is much less than the external field. Strictly speaking, the uniform demagnetizing field only occurs in homogeneous bodies whose shape has the form of an ellipsoid, and it is non-uniform in a rectangular specimen, which is also heterogeneous. However, in the practical applications of magnetism, Equation (1) is commonly used for the estimation of the effective internal field *H*_in_ in thin-plate samples.

The influence of demagnetization effects on the ME characteristics of conventional layered composites made of continuous materials has been considered before in [21,22]. Boucher et al. [23] investigated excitation of ferromagnetic resonance modes in an array of ferromagnetic nanowires. They came to the conclusion that, in that case, both the intra-wire shape demagnetizing field and the extra-wire shape demagnetizing field (the interaction field) were important for describing their experimental results. 

Figure 3 also demonstrates that the magnitude of the magnetic anisotropy was influenced by the distance between the Ni-wires. It can be seen that when magnetized along the wires (Curve 1), the saturation fields for both samples were approximately the same. When magnetization occurred in the transverse direction (Curve 2), the saturation field *H*_S_ was smaller and the slope of the *M*(*H*) curves in small fields was larger for the MFC with a period of 250 μm than for the MFC with a period *T*_2_ of 500 μm. This behavior can be explained by the dependence of the magnetic properties on the concentration of the ferromagnetic phase in a composite material. According to [23], the effective field, acting upon the magnetization of the individual wires in the transverse direction, grows with the increasing concentration of FM wires in a composite material. In [18], a steep rise in the effective transverse magnetic permeability was revealed near the percolation threshold. By selecting the length, the diameter of the FM wire, and the distance between them, one can obtain a magnetization curve in the transverse direction, the course of which is intermediate between the limiting cases: The magnetization curve of an in-plane magnetized thin homogeneous FM layer and the curve of a single transversely magnetized FM rod. It is significant that at the same time, the magnetization curves of an MFC along Ni-wires do not change much. 

In a similar way, geometrical anisotropy led to the dependence of the shape of the magnetostriction curves *λ*(*H*), shown in Figure 4, on the direction of ***H***. When magnetized along the axis of Ni-wires, the magnetostriction of both MFC samples was saturated in approximately the same field *H*_S_ ≈ 600 Oe. The saturation level of the magnetostriction *λ*_S_ for the MFC was lower than for the continuous Ni layer. Moreover, *λ*_S_ was smaller for the larger distance *T* between the wires and, accordingly, the lesser fraction of the magnetostrictive material in the MFC composition. With a decrease in *λ*_S_ of the composite, the value of the piezomagnetic module obviously decreased as well, $\lambda^{\left(1\right)}(H)=\partial \lambda /\partial H∼\lambda_{s}/H_{s}^{}$.

As can be seen in Figure 4, when an MFC was magnetized in the transverse direction, the magnetostriction was saturated in larger fields. This led to a significant reduction in the piezomagnetic modulus of the MFC. The shape of the magnetostriction curve *λ*(*H*) and the shape of the $\lambda^{\left(1\right)}(H)$-dependence for the MFC could also be changed by selecting the diameter of the wires and the distance between them.

Let us estimate the frequencies of acoustic resonances of the considered MFC-PZT structure. For a homogeneous, free-standing thin square-shaped plate, the frequencies of the lowest modes of flexural *f*_1_ and longitudinal *f*_2_ vibrations are given by the Equations (2) [24]:

(2) $$f_{1}=\frac{8ak_{1}}{\pi L^{2}}\sqrt{\frac{Y}{12\rho (1-\gamma^{2})}},\;\mathrm{and}\;f_{2}=\frac{1}{2L}\sqrt{\frac{Y}{\rho (1-\gamma^{2})}},$$

where $k_{1}$ = 14.1 is the numerical coefficient for the lowest flexural oscillation mode, *a* is the plate thickness, *L* is the side length, *Y* is the Young’s modulus, *ρ* is the density, and γ is the Poisson’s ratio. 

For a composite MFC-PZT structure, the effective Young’s modulus and the effective density are estimated as:

(3) $$Y_{}=\sum_{i}Y_{i}S_{i}/\sum_{i}S_{i}\mathrm{and}\rho =\sum_{i}\rho_{i}S_{i}/\sum_{i}S_{i}.$$

Here, *S*_i_ is the cross-section of all Ni-wires, polymer matrix, and PZT layer, respectively. The total thickness *a* of the structure was equal to 405 μm, the length *L* was equal to 10 mm, and the parameters of the materials were: Nickel—*Y*_1_ = 21.5 × 10^10^ N/m^2^, *ρ*_1_ = 8.8 × 10^3^ kg/m^3^; polymer—*Y*_2_ = 0.22 × 10^10^ N/m^2^, *ρ*_2_ = 1.13 × 10^3^ kg/m^3^; and PZT—*Y*_3_ = 7 × 10^10^ N/m^2^, *ρ*_3_ = 7.7 × 10^3^ kg/m^3^. The Poisson’s ratio *γ* was taken to be equal to 0.3. The substitution of the parameter values corresponding to the MFC-PZT structure with a period *T*_1_ of 250 μm into Equations (2) and (3) gave the bending vibration frequency *f*_1_ ≈ 42.7 кHz and the planar vibration frequency *f*_2_ ≈ 203 kHz. The calculated frequency of bending vibrations *f*_1_ reasonably agreed with the measured value. The deviation can be attributed to a neglect of the anisotropy of mechanical properties of a composite material in Equation (2). 

Using the data in Figure 5, let us estimate the maximum value of the ME coefficient for the MFC-PZT structure at the resonance frequency. When the structure was magnetized along the Ni-wire, we obtained $\alpha_{E∥}=u/(a_{p}h)$ ≈ 4.71 V/A, and when the magnetization was perpendicular to the wires, we got $\alpha_{E⊥}$≈ 0.23 V/A. Thus, the ME coefficient for MFC-PZT structures is comparable with the typical value $\alpha_{E}$~ 1–10 V/A for Ni-PZT structures with a continuous nickel layer [25,26]. Using the data in Figure 10 and Figure 11, let us estimate the maximum efficiency of second harmonic generation in the MFC-PZT structure with a period *T*_1_ = 250 μm: $\alpha_{E}^{\left(2\right)}=u_{2}/(a_{p}h^{2})$ ≈ 1.1 mV∙m/A^2^. As far as the order of magnitude is concerned, the obtained value is consistent with the efficiency of frequency doubling in the Ni-PZT structure comprising a continuous nickel layer [14].

The shapes of the field dependence of the ME voltage *u*_1_(*H*) in Figure 5 and the field dependence of the second harmonic *u*_2_(*H*) in Figure 9 were determined by the field dependence of the MFC magnetostriction *λ*(*H*). It has been theoretically shown that the amplitudes of the first and second harmonics of the ME voltage, generated by a composite structure at the frequency of acoustic resonance, are given by the following expressions of Equation (4)

(4) $$u_{1}=AQd_{31}\lambda^{\left(1\right)}h\;\mathrm{and}\;u_{2}=AQd_{31}\lambda^{\left(2\right)}h^{2},$$

respectively [27]. Here *A* is a coefficient depending on the size and mechanical parameters of the layers of the structure, *Q* is the quality factor of an acoustic resonance, $d_{31}$ is the piezoelectric coefficient, $\lambda^{\left(1\right)}=\partial \lambda /\partial H=q_{11}$ is the linear piezomagnetic coefficient (the first derivative of magnetostriction with respect to the field), $\lambda^{\left(2\right)}=\partial^{2}\lambda /\partial H^{2}$ is the nonlinear piezomagnetic coefficient (second derivative of the magnetostriction with respect the field), and *h* is the amplitude of the excitation magnetic field. 

The measured *λ*(*H*) dependence shown in Figure 4, was empirically approximated by the following equation:

(5) $$\lambda (H)=\lambda_{s}\left(1-\exp\left[-{\left(H/G(H)\right)}^{2}\right]\right)$$

where the fitting parameters are: *λ*_s_ = 35 × 10^−6^ and $G(H)=\left\{7162+0.69\left|\left(H\cdot 1\;m/A\right)\right|+1.257\cdot 10^{-7}\cdot {\left(H\cdot 1\;m/A\right)}^{2}\right\}\times 1\;A/m$. Equation (5) gives a quadratic dependence *λ* ~ *H*^2^ in the small-field region and describes the experiment in the intermediate-field region and the magnetostriction saturates at the level of *λ*_S_ in the region of large fields. Furthermore, the first and second derivatives of this function have been found by means of numerical differentiation. For comparison with experiments, the calculated field dependence $\lambda^{\left(1\right)}(H)$ is shown by a solid line in Figure 6a, and the calculated dependence $\lambda^{\left(2\right)}(H)$ is given in Figure 10. A good agreement between the shape of the measured and calculated dependencies confirmed the possibility of using the existing theory (Equation (4)) to describe the ME characteristics of planar structures with MFC layers.

Finally, we note that anisotropic ME effect in the structures with MFC and PE materials can be used to design sensors of DC magnetic fields, which allows one to determine field orientation. At the same time, a sufficiently large area of the PE layer enables a significant magnitude of the output signal, that is, a high responsivity of the sensor. The use of an MFC combined with a PE layer made of a PE polymer (for example, PVDF (polyvinylidene fluoride)) or with a PFC-composite will make it possible to create flexible ME sensors that can be applied to curved surfaces. It is of interest to employ flexible structures with MFCs to design actuators that possess large displacement amplitudes controlled by a magnetic field. 

## 5. Conclusions

To summarize, the planar structures containing a magnetostrictive fibrous composite and a piezoelectric layer, mechanically bonded to each other, were fabricated. The MFC was an array of FM (nickel) wires that were ordered in a single layer parallel to each other and embedded in a polymer matrix. It is shown that the magnetization and magnetostriction of the MFC strongly depend on the orientation of the magnetizing field ***H*** and the distance between the Ni-wires. This leads to a pronounced anisotropy of the direct ME effect in MFC-PZT structures resulting from the combination of magnetostriction and piezoelectricity. The ME coupling coefficient in the proposed heterostructure (about 4.8 V/A) is comparable with the ME coefficient in composite structures with a continuous nickel layer. The large anisotropy of the ME coupling coefficient (the ratio of the value along the wires to the value in the perpendicular direction is about 20) is promising for the development of sensors of DC magnetic fields, which would allow one to determine field orientation. With an increase in the excitation field, the generation of the second harmonic of the ME voltage at the frequency of the flexural vibrations of the structure has been observed. It is shown that the characteristics of the ME transduction in the MFC-PZT structure are well described by the existing theory.
